# Association of Nerve Conduction Study Variables with Hematologic Tests in Patients with Type 2 Diabetes Mellitus

**DOI:** 10.3390/medicina61030430

**Published:** 2025-02-28

**Authors:** Jung-Eun Han, Jun-Hwan Choi, So-Yeon Yoo, Gwan-Pyo Koh, Sang-Ah Lee, So-Young Lee, Hyun-Jung Lee

**Affiliations:** 1 Department of Internal Medicine, Jeju National University Hospital, Jeju National University College of Medicine, Jeju 63241, Republic of Korea; heather920@naver.com (J.-E.H.); happyweed@jejunu.ac.kr (S.-Y.Y.); okdom@jejunu.ac.kr (G.-P.K.); sahe7@hanmail.net (S.-A.L.); 2 Department of Rehabilitation Medicine, Jeju National University Hospital, Jeju National University College of Medicine, Jeju 63241, Republic of Korea; bluelsy900@hanmail.net (S.-Y.L.); sigano@hanmail.net (H.-J.L.)

**Keywords:** diabetic neuropathy, nerve conduction study, hematologic test

## Abstract

*Background and Objective*: Diabetic peripheral neuropathy (DPN) is a prevalent complication of type 2 diabetes mellitus (T2DM), with nerve conduction studies (NCSs) serving as the diagnostic gold standard. Early diagnosis is critical for effective management, yet many cases are detected late due to the gradual onset of symptoms. This study explores the relationship between hematological tests and NCS outcomes in T2DM patients to improve the early detection of DPN. *Material and Methods*: This retrospective study involved T2DM patients exhibiting neuropathic symptoms, and patients were divided based on NCS findings into groups with normal and abnormal results to assess the diagnostic value of various hematological markers, clinical, and demographic data for DPN. *Results*: Among 400 participants, 57% (*n* = 228) had abnormal NCS results indicative of DPN. Significant differences were observed in the abnormal-NCS group, including older age, longer diabetes duration, higher levels of fasting plasma glucose, HbA1c, and apolipoprotein B, along with lower eGFR, HDL-C, and Apo A-I levels. Notably, negative correlations were found between HDL-C, Apo A-I, vitamin B12, and specific NCS measurements, while positive correlations existed with sural sensory nerve amplitudes. Multivariate analysis highlighted the importance of age, diabetes duration, hyperglycemia, and specific hematologic markers in predicting DPN. *Conclusions*: The findings confirm that NCSs, combined with hematologic testing, can effectively identify DPN in T2DM patients. Consistent with prior research, prolonged hyperglycemia and nephropathy progression are strongly linked to DPN development. Additionally, lower levels of HDL-C, Apo A-I, and vitamin B12 are associated with the condition, suggesting their potential utility in early diagnostic protocols.

## 1. Introduction

Diabetic peripheral neuropathy (DPN) is one of the most common microvascular complications of type 2 diabetes mellitus (T2DM), with a prevalence of approximately 50% or more of patients with T2DM [1]. Typical DPN is a symmetrical, length-dependent sensorimotor polyneuropathy caused by metabolic and vascular changes resulting from long-term exposure to hyperglycemia and cardiovascular risk covariates [2]. However, compared with the effect of hyperglycemia treatment on DPN in type 1 diabetes mellitus [3,4], the correlation between DPN and hyperglycemia in patients with T2DM remains unclear. The UK Prospective Diabetes Study found that ten years of glycemic treatment reduced the incidence of overall microvascular complications; however, the reduction in DPN alone was uncertain [5], and the Action to Control Cardiovascular Risk in Diabetes (ACCORD) study showed that intensive glycemic control had no significant effect on the incidence of DPN [6].

The exact pathogenesis is unknown, and experimental studies suggest multifactorial causes and pathogenic pathways driven by oxidative and inflammatory stress, hyperglycemia, and other metabolic factors such as hyperlipidemia and insulin resistance [7,8]. The polyol, glycation, protein kinase C, poly (ADP-ribose) polymerase (PARP), and hexosamine pathways, which are known to cause oxidative stress in neurons and microvessels, are specifically affected by hyperglycemia [9,10]. However, as the previous studies mentioned above could not reduce the occurrence of DPN with blood sugar control alone, many other factors are thought to be involved.

Early diagnosis is vital to prevent the progression of DPN and to manage it properly; however, in the early stages of T2DM, DPN is an insidious and asymptomatically progressing condition [11,12] and no consensus regarding early diagnosis exists [11]. Therefore, therapeutic interventions are often postponed. The diagnosis of DPN usually depends on clinical symptoms and signs of neuropathy, including pain, tingling, and numbness. However, these methods for diagnosing DPN have disadvantages, such as limited sensitivity and high variability [13]. Nerve conduction studies (NCSs) have long been known as the gold standard test for DPN diagnosis and the detection of nerve damage [14]. Although damage to small sensory nerve fibers is one of the earliest manifestations of DPN [13], because it only assesses damage to large fibers, the findings are often separate from the subjective symptoms of DPN. In addition, an NCS requires special equipment and skilled inspectors and, therefore, has moderate reproducibility.

Previous studies have revealed that abnormalities in NCSs are related to glycemic control, DM duration, age, male sex, and height [15,16,17,18]. However, there is little evidence of an association between abnormalities in NCSs derived from DPN and blood markers [19,20]. Therefore, this study aimed to investigate the association between hematologic tests and NCS variables in patients with T2DM.

## 2. Materials and Methods

### 2.1. Study Design and Participants

This retrospective study was conducted using the medical records of patients with T2DM who visited the Department of Endocrinology at Jeju National University Hospital between March 2011 and June 2019 for evaluation of complications. Among the patients whose medical records were reviewed, those who were referred for NCS examination because of suspected neuropathic symptoms were included.

The exclusion criteria were as follows: (1) focal entrapment results such as carpal and cubital tunnel syndromes, (2) medical conditions (e.g., thyroid disease, malignant neoplasm, and vasculitis), and (3) exposure to toxins or medicines that could cause peripheral neuropathy (e.g., alcohol and neurotoxic chemotherapy).

All patients had NCSs and hematologic tests ordered by the endocrinologists, and both examinations were performed within a week.

### 2.2. Electrophysiologic Studies

Eligible patients underwent a standard NCS using a Medelec Synergy electromyography machine (Medelec Synergy, Oxford, UK) with surface electrode recording and stimulation. Electrodes were applied to both legs and the right arm. The temperature of the NCS examination room was maintained between 22 °C and 24 °C, and the skin temperature was above 32 °C and 30 °C for the upper and lower limbs, respectively. All the NCS assessments were performed by a well-trained technician.

The conduction velocity, amplitude (from baseline to peak), and distal latency of compound muscle action potentials (CMAPs) were obtained in the median, ulnar, peroneal, and tibial nerves using the orthodromic technique. The CMAPs were measured using a 3–10,000 Hz filter, 5 ms/division sweep speed, and 5 mV/division sensitivity. The distal latency and amplitude (from baseline to peak) of the sensory nerve action potentials (SNAPs) in the median, ulnar, sural, and superficial peroneal nerves were measured using an antidromic technique. The SNAPs were performed using a 20–2000 Hz filter, 1 ms/division sweep speed, and 20 µV/division sensitivity. The latency of the F-waves in the median, ulnar, peroneal, and tibial nerves and the Hoffmann reflex (H-reflex) of the tibial nerve were recorded. The F-wave and H-reflex were measured by a 20–10,000 Hz filter, 5 ms/division sweep speed, and 500 µV/division sensitivity, and a 30–10,000 Hz filter, 10 ms/division sweep speed, and 500 µV/division sensitivity, respectively.

DPN diagnosis was determined based on the presence of at least two abnormalities in the aforementioned NCSs. DPN severity was classified into the following stages: mild (two or more abnormalities in the H-reflex [21], tibial F-wave [22], or sural SNAP results [23]), moderate (mild stage with two or more abnormalities on SNAP in the upper extremity nerves or CMAP in the lower extremity nerves), and severe (two or more abnormalities on CMAP or F-waves in the upper extremities).

### 2.3. Hematologic Tests

All blood samples were collected in the morning, following a fast beginning at midnight. Plasma glucose was measured by the hexokinase-glucose-6-phosphate method, total cholesterol (TC) was analyzed by the enzymatic colorimetric method, triglyceride (TG) was analyzed by the glycerol-3-phosphate (GPO)–peroxidase (POD) chromogenic method, high-density lipoprotein cholesterol (HDL-C) and low-density lipoprotein cholesterol (LDL-C) were analyzed by the direct enzymatic method, apolipoprotein A1 (Apo A1) and B (Apo B) were measured by an immunoassay (TIA) using an automatic chemical analyzer (TBA-FX8, Toshiba, Tokyo, Japan). Vitamin B12 levels were analyzed using a Chemiluminescent Microparticle Immunoassay (CMIA).

The urine albumin–creatinine ratio (UACR) was measured using an automatic chemical analyzer (Hitachi 7600-110, High-Tech, Tokyo, Japan).

The following formula was used to determine the glycemic exposure (GE) index using HbA1c and diabetes duration [24]:

Glycemic exposure (GE) index = (HbA1c)^1/2^ × (duration of DM in years)^1/8^

### 2.4. Statistical Analysis

All variables were analyzed using descriptive statistics. Differences in demographics, disease-related traits, and laboratory investigations were compared using Student’s *t*-test. Pearson correlation coefficients were used to evaluate the relationships between laboratory studies and the latencies of the H-reflex, tibial F-wave, and amplitude of sural SNAP. Multivariate analysis of variance (MANOVA) was used to determine the relationship between DPN severity and laboratory results. Independent factors and the presence of DPN were determined using multivariate analysis and binary logistic regression. The optimal cut-point selection of variables was determined by the maximum value of the Youden index in the ROC curve analysis among the significant variables in MANOVA. All statistical analyses were performed using IBM SPSS Statistics for Windows version 22.0 (SPSS Inc., Armonk, NY, USA) and MedCalc Version 22.023 (Medcalc software Ltd., Ostend, Belgium). A *p*-value of <0.05 was considered statistically significant.

## 3. Results

**Figure 1:**
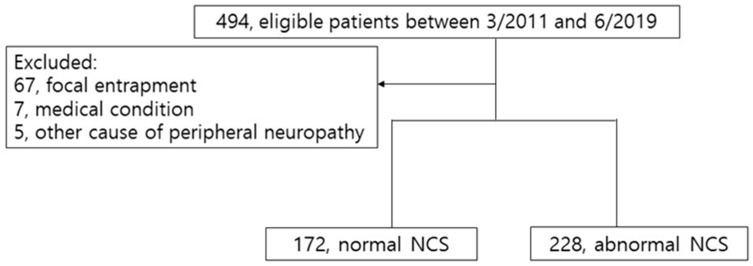
Flow chart of this study.

A total of 494 patients were referred for DPN evaluation during the study period. Among these patients, 94 met the exclusion criteria (Figure 1). The final analysis included 400 patients (173 male and 227 female) with a mean age of 58.7 ± 14.8 and a mean diabetes duration of 11.5 ± 8.3 years. The NCSs revealed abnormal values in 57% (*n* = 228) of the patients, and 125 (25.3%), 123 (25.3%), and 74 (15%) patients were in the mild, moderate, and severe stages, respectively.

The demographic and disease-related characteristics and hematological test results of the patients are presented in Table 1. The mean age, DM duration, HbA1c level, GE index, fasting plasma glucose (FPG) level, Apo B level, and UACR were higher in the abnormal-NCS group. HDL-C, Apo A1, estimated glomerular filtration ratio (eGFR), and vitamin B12 levels were higher in the normal-NCS group.

Table 2 shows the correlation between the demographic, disease-related characteristics, laboratory results, H-reflex latency, tibial F-wave latency, and sural SNAP amplitude. Older age and long-term exposure to hyperglycemia were positively correlated with delayed H-reflex and tibial F-wave latencies and negatively correlated with sural SNAP amplitude. HDL-C, Apo A1, and vitamin B12 levels had significant negative correlations with the latencies of the H-reflex and tibial F-wave and negative correlations with the amplitude of the sural SNAP.

In the multivariate binary logistic regression analysis, older age, higher GE index, HbA1c, and UACR, and lower Apo A1 and eGFR were significantly associated with DPN (Table 3).

In this study, we conducted a subgroup analysis of the correlations based on the NCS results. In patients with normal NCS results, HbA1c showed a significant positive correlation with bilateral tibial F-wave latency (r = 0.190/0.248, *p* < 0.001), HDL-C was negatively correlated with bilateral tibial F-wave latency (r = −0.284/−0.254, *p* < 0.001), and Apo A1 was negatively correlated with bilateral H-reflex latency (r = −0.732/−0.382, r < 0.001) and tibial F-wave latency (r = −0.229/−0.261, *p* < 0.001). The eGFR and UACR were positively correlated with the bilateral amplitude of sural SNAP (r = 0.256/211 and 0.329/0.291, respectively; all *p* < 0.001). In the subgroup analysis of abnormal NCS results, DPN severity was related to the UACR in the MANOVA.

## 4. Discussion

This study investigated the relationship between hematological tests and NCS results in patients with clinical DPN. The abnormal-NCS group showed poor glycemic control, dyslipidemia, and decreased renal function compared to the patients with normal electrophysiological studies.

The results of this study showed that poor blood sugar control and the period of exposure to hyperglycemia worsened DPN risk and severity. These results are similar to those of hyperglycemia, which was the main cause of neuropathy in patients with type 2 diabetes in previous studies [25,26]. Although hyperglycemia is known to be an important etiology of DPN, glycemic control did not reduce DPN risk in patients with T2DM in large-scale studies such as the ACCORD trial [6]. However, this delayed its occurrence. Therefore, it is thought that the occurrence of DPN in T2DM patients is not only caused by hyperglycemia but also involves various metabolic factors. Previous studies have indeed identified dyslipidemia and insulin resistance as relevant hematological findings [20,25]. However, there are limited studies on other hematological markers. Therefore, it is necessary to determine the relationship between hematological tests for T2DM and neuropathy. Therefore, prolonged hyperglycemia is still considered one of the most critical etiological factors of DPN.

The American Academy of Neurology, American Association of Electrodiagnostic Medicine, and American Academy of Physical and Rehabilitation defined DPN as a symmetrical, length-dependent sensorimotor polyneuropathy caused by metabolic causes such as hyperglycemia and microvessel alteration [14]. In addition, an NCS is the gold standard for confirming nerve damage, and the NCS for diagnosing DPN is the most objective and widely used worldwide [2]. In this study, assuming that diabetic neuropathy is length-dependent, we analyzed the H-reflex, F-wave, and sural SNAP test results, which are thought to be the first abnormal findings in NCS DPN results. The H-reflex is a monosynaptic reflex arc arising from the Ia afferent activation of muscles to the alpha motor neurons [27]. Therefore, this is one of the evaluations of the longest nerve pathways in the NCS. The H-reflex could have a predictive value in early DPN diagnosis [28] and the F-wave latency of the tibial nerve is one of the most sensitive measures in an NCS to detect subclinical or overt DPN [22,29]. Sural nerve conduction study amplitude is a reliable method for diagnosing mild DPN [12].

Based on its reproducibility, reasonable sensitivity, and specificity, NCSs have been used as an endpoint in clinical trials on human diabetic neuropathy to measure large myelinated nerve fiber function [30]. Therefore, this study analyzed the correlation between changes in the H-reflex, tibial F-wave, and sural nerve amplitude, which are the initial findings of NCS abnormalities in patients with DPN among those with normal NCS test results. However, this study was based on the NCS results of patients clinically thought to have DPN, and it cannot be concluded that abnormal findings in the NCS results indicate that the neuropathy has progressed further. Since the subjects were patients clinically suspected of having neuropathy, it cannot be said that neuropathy does not exist even if the NCS results are normal. Because approximately 80–90% of peripheral nerves are small fibers [12,31], several studies have shown that small sensory nerve fibers are one of the earliest manifestations of DPN [32,33,34]. However, when referring to studies showing that DPN progresses from damage to small fibers to damage to large fibers, abnormal findings on the NCS can be considered to be slightly more advanced DPN. Although the results of the NCS are within the normal range of the diagnostic criteria, it would be helpful for early diagnosis if changes in the NCS could be identified more quickly.

Dyslipidemia, especially low HDL-C levels, is a noteworthy phenomenon. In this study, HDL-C and its precursor Apo A1 were statistically correlated with the latency of the H-reflex, tibial F-wave, and sural nerve amplitude. Additionally, the logistic regression analysis demonstrated that the likelihood of receiving a DPN diagnosis in the NCS increased with decreasing Apo A1 levels, a precursor to HDL. Additionally, logistic regression analysis showed that the probability of DPN diagnosis in the NCS increased with decreasing Apo A1 levels. However, no statistically significant results were observed for LDL or its precursor Apo B. Considering that all patients in this study had DPN, HDL may play a role in preventing exacerbations. A possible explanation is that in diabetes animal models, HDL-C and Apo A1 improve insulin sensitivity and pancreatic β-cell survival and function, which in turn improves glycemic control [35].

In addition to treating hyperlipidemia with drugs, a strategy to increase HDL levels is needed. These results showed no significant differences in the TC, TG, and LDL levels between the two groups, which is thought to be because most patients were receiving treatment for hyperlipidemia. Current study showed no discernible changes in TC, TG, or LDL levels between the two groups, which is assumed to be because most patients were receiving treatment for hyperlipidemia. Drugs that lower LDL and TG levels, such as niacins, fibrates, and statins, occasionally increase HDL levels. However, large-scale randomized controlled trials of interventions for HDL-C levels have not responded favorably to epidemiological evidence of an inverse relationship between HDL-C levels and the risk of atherosclerotic cardiovascular disease [36]. Therefore, physical activity, weight loss, diet, and smoking cessation are important factors that increase HDL-C levels.

Significant negative correlations were found between H-reflex latency, tibial F-wave, kidney function, and vitamin B12. Diabetic nephropathy is a major complication of diabetes and is associated with long-term exposure to high blood sugar levels and other metabolic or hemodynamic problems [37]. In this study, increased microalbumin levels and decreased renal function, which are early indicators of nephropathy, appeared to be associated with DPN occurrence. This suggests that neuropathy must be identified in the early stages. However, the prevalence of nephropathy and neuropathic complications is not well established. In addition, the relationship between the severity of nephropathy and neuropathy, as well as that between the degree of nephropathy progression and neuropathy occurrence, is not well known.

In addition, several studies have shown that the administration of vitamin B12 improves DPN symptoms [38,39], and studies have shown that a decrease in vitamin B12 increases DPN occurrence [40]. Similar to the aforementioned studies, vitamin B12 levels were lower in patients with abnormal NCS results who were thought to have more advanced neuropathy. In the NCS results, lower vitamin B12 levels increased H-reflex and F-wave latencies and decreased the sural nerve amplitude. Binary logistic regression analysis showed that lower vitamin B12 levels were associated with an increased risk of DPN diagnosis based on NCS.

This study has some limitations. First, there is no established NCS protocol for diagnosing DPN. Although it was conducted based on the literature, additional research is needed to determine whether the changes in the H-reflex, F-wave, and Sural nerve, which were assumed to be initial changes in NCS in this study, can truly represent changes in the initial DPN. Second, this was a retrospective study, and the participants were clinically presumed to be patients. Therefore, participants could not be evaluated using screening or assessment tools. Future studies should use additional quantitative assessment tools. Finally, this study only included patients with T2DM who had been diagnosed with diabetes for a relatively long period. Therefore, a prospective study that can confirm these changes in patients with early-stage diabetes is necessary.

## 5. Conclusions

This study demonstrated that NCS can be used to identify hematological indicators helpful in diagnosing DPN. Patients with T2DM with long-term exposure to hyperglycemia and progression of nephropathy are thought to be associated with the development of DPN. It is necessary not only to control hyperglycemia but also to assess metabolic factors such as HDL-C, Apo A-I, and vitamin b12 to prevent DPN. Glycemic control alone is insufficient to prevent the progression and worsening of DPN. Additionally, lower levels of HDL-C, Apo A-I, and vitamin B12 are associated with the condition, suggesting their potential utility in early diagnostic protocol. Therefore, this study is clinically meaningful in identifying the risk of developing DPN early through hematological testing and confirming it through NCS.

## Figures and Tables

**Table 1:** Demographic, disease-related characteristics, and laboratory studies of the subjects (*n* = 400).

Variables	Normal NCS (*n* = 172)	Abnormal NCS (*n* = 228)	*p*-Value
Age (years)	54.5 ± 14.3	62.1 ± 13.7	<0.001 **
Sex, males/females	81 (47.1)/91 (52.9)	92 (40.4)/136 (59.6)	0.186
BMI	25.1 ± 4.4	25.6 ± 4.7	0.299
DM duration (years)	8.6 ± 6.6	13.6 ± 9.0	<0.001 **
HbA1c (%)	8.4 ± 2.3	9.2 ± 2.6	<0.001 **
GE Index	4.4 ± 3.4	7.7 ± 5.8	<0.001 **
FPG (mg/dL)	172.9 ± 83.9	198.8 ± 116.4	0.011 *
C-peptide (nmol/L)	2.18 ± 1.4	2.36 ± 1.8	0.265
TC (mg/dL)	156.4 ± 64.0	164.9 ± 44.7	0.135
TG (mg/dL)	148.1 ± 141.7	158.1 ± 232.2	0.445
HDL-C (mg/dL)	50.4 ± 19.9	44.1 ± 16.6	<0.001 **
Apo A1 (mg/dL)	63.7 ± 70.7	40.2 ± 63.3	0.001 **
LDL-C (mg/dL)	84.3 ± 38.7	88.4 ± 34.3	0.239
Apo B (mg/dL)	31.5 ± 50.5	49.9 ± 57.9	0.001 **
eGFR	95.8 ± 69.7	79.6 ± 30.5	0.009 *
UACR	62.5 ± 171.3	299.0 ± 828.8	<0.001 **
Vitamin B12	752.1 ± 380.7	632.2 ± 358.3	0.003 **

Values represent mean ± standard deviation or number (%) of cases. Abbreviations: NCS, nerve conduction study; DM, diabetes mellitus; FPG, fasting plasma glucose; HbA1c, glycosylated hemoglobin; GE Index, glycemic exposure index; TC, total cholesterol; TG, triglyceride; HDL-C, high-density lipoprotein cholesterol; Apo, apolipoprotein; LDL-C, low-density lipoprotein cholesterol; eGFR, estimated glomerular filtration rate; UACR, urine albumin–creatinine ratio; * < 0.05, ** < 0.01.

**Table 2:** Correlation between latency of NCS and disease-related characteristics in the laboratory studies.

Variables	Coefficients (r) H-Reflex (Rt/Lt)	Coefficients (r) Tibial F-Wave (Rt/Lt)	Coefficients (r) Sural Amp (Rt/Lt)
Age (years)	0.196 **/0.217 **	0.201 **/0.131 **	−0.397 **/−0.376 **
BMI	0.118/0.147	0.052/0.015	−0.028/0.043
DM duration	0.217 **/0.179 *	0.223 **/0.163 **	−0.299 **/−0.309 **
GE Index	0.221 **/0.177 **	0.264 **/0.195 **	−0.318 **/−0.319 **
HbA1c (%)	0.093/0.076	0.204 **/0.230 **	−0.105/−0.084
FPG (mg/dL)	0.146/0.157	0.120 */0.146 **	−0.099/−0.058
C-peptide (nlmol/L)	0.064/0.089	−0.002/0.049	−0.065/−0.046
TC (mg/dL)	−0.058/−0.021	−0.065/−0.009	0.163 */0.159 *
TG (mg/dL)	0.096/0.118	0.003/0.108	0.055/0.055
HDL-C (mg/dL)	−0.152 **/−0.180 **	−0.202 **/−0.183 **	0.141 */0.160 *
Apo A1 (mg/dL)	−0.373 **/−0.195 *	−0.231 **/−0.238 **	0.214 */0.278 **
LDL-C (mg/dL)	−0.040/−0.001	0.034/−0.014	0.103/0.106
Apo B (mg/dL)	−0.030/−0.121	−0.004/−0.009	−0.033/0.041
eGFR	−0.057/0.022	−0.088/−0.027	0.262 **/0.216 *
UACR	0.132/0.195 *	0.173 **/0.205 **	−0.163 */−0.204 *
Vitamin B12	−0.205 **/−0.246 **	−0.113 */−0.125 *	0.261 **/0.168 *

NCS, nerve conduction study; Rt, right; Lt, left; DM, diabetes mellitus; GE Index, glycemic exposure index; FPG, fasting plasma glucose; HbA1c, glycosylated hemoglobin; TC, total cholesterol; TG, triglyceride; HDL-C, high-density lipoprotein cholesterol; Apo, apolipoprotein; LDL-C, low-density lipoprotein cholesterol; eGFR, estimated glomerular filtration rate; UACR, urine albumin–creatinine ratio. * < 0.05, ** < 0.01.

**Table 3:** Multivariate analysis using binary logistic regression of laboratory studies and disease related characteristics for DPN.

Variables	Logistic Regression		
	Odds Ratio	95% CI	*p*-Value
Age > 62.5	2.582	1.708–3.902	<0.001
GE index > 4.78	3.629	2.386–5.519	<0.001
HbA1c > 7.75	2.195	1.461–3.299	<0.001
ApoA1 < 100	2.003	1.315–3.050	0.001
eGFR < 62.65	7.969	3.457–18.370	<0.001
UACR > 37.465	4.250	2.700–6.691	<0.001
Vitamin B12 < 520	1.684	1.041–2.722	0.034

GE Index, glycemic exposure index; HbA1c, glycosylated hemoglobin; Apo, apolipoprotein; eGFR, estimated glomerular filtration rate; UACR, urine albumin–creatinine ratio.

## Data Availability

Data are available upon request.
